# Therapeutics

**Published:** 1876-08

**Authors:** 


### II. Therapeutics.

1. *The Electrolytic Treatment of Tumors.* Julius Althans. (London.) *Berl. Kl. Wochenschr.*, Allg. Med. Central. Zt., 35, 1876.

In consideration of the great difference in opinions about the therapeutic usefulness of electrolysis, the author gives a brief account of his latest observations in this field.

1. *Nævus.* It is most frequently found in the face and on the scalp, and electrolysis is the most appropriate means for its removal. It is superior to excision, because there is no hemorrhage; to injections of sesquichloride of iron, because it does not endanger the life; to the cauterization by nitric acid, because it can be localized better; to the subcutaneous ligation, because after the operation the child has no pain and the surgeon has no further trouble; over the galvanocaustic, because it does not leave any scar. The ordinary round, small and thin nævus usually yields to one single application, while the larger "portroine stains" require a number of sittings.

The current acts through needles, which set in rows, and connected with the poles of ten to fifteen Daniell's elements are thrust into the tissue of the naevus. As soon as the chain is closed the destruction of the texture begins with the blood vessels and the integument of the nævus appearing to wither. The destruction being more thorough around the positive pole, this must always be applied to the worst portion of the nævus. Usually not a single drop of blood is lost; if, however, by any sudden motion of the patient, a needle should come out, and a drop of blood should appear, this can be coagulated at once and any further bleeding can be prevented by directing the positive pole to the puncture. When all the morbid tissue seems to be destroyed, the current is interrupted and the surface of the nævus is covered by a piece of gold beater's skin. No further dressing is necessary because the surface remains dry. The eschar is thrown off after about ten or fourteen days, and the surface gradually assumes the appearance of the neighboring integument.

2. *Goitre.* The writer considers electrolysis as especially applicable to the cystic goitre, because it occasions neither pain nor danger. The best *modus operandi* is to introduce two or three needles into the cyst and to connect the same with the negative pole, while the positive electrode is applied to the integument by means of a wet sponge. Through the electrolytic action caustic soda is created out of the solution of chloride of sodium contained in the cyst. This caustic soda cauterizes the secretory lining of the cyst and stops any further secretion. Two to six applications usually are sufficient. In the treatment of parenchymatous goitre the author combines the electrolysis with injections of tincture of iodine into the tissue of the goitre. These parenchymations injections break up the texture of the tumor and thus the combined method shortens the time of treatment quite materially, especially if the goitre is old and rigid.

3. *Sebaceous Tumors.* They can easily be excised; but many patients dreading an operation, and the excision being occasionally followed by dangerous, even fatal, erysipelas, Althaus advises the use of electrolysis, which will remove these tumors without danger and without leaving a scar. Both electrodes are put into the tumor to have a quick result.

4. *Carcinoma.* The writer advocates the early and thorough extirpation of every primary cancer. But in cases of secondary carcinomata the electrolysis has often done a good service. It cannot, of course, eradicate the cancerous diathesis, nor prevent the fatal termination, but it is very efficient in allaying those lancinating pains, in procuring sleep and comfort to the patient.

The histories of cases are given in support of the author's views expressed in the paper.

2. *Diphtheria Treatment.* Z. C. McElroy, M. D. (*W. Va. Med. Student,* May, 1876.)

This medical philosopher, always sensible and somewhat iconoclastic at times, thus treats this dreaded disease:

"For the bulk of cases, where the temperature does not remain high, or does not reach higher than 103° or 104° F., I seldom make more than one prescription, and am not often called to supplement it by other measures, except in severe cases, by some opiate to relieve headache, or something to open the bowels. In the latter case I do so by half a grain calomel granules, one every six hours, the desired evacuation almost always occurring within twenty-four hours. The prescription almost always used for an adult is about as follows:

| | | |
|---|---|---|
| ℞ | Chlorate Potass | ʒj. |
| | Dilute Alcohol, } *aa.* Pure Water, } | fʒvj. |
| | Tinct. Capsicum | fʒiij. |
| | Liq. Ferri Perchlor. | fʒj. |

Sig.—A teaspoonful in a wine-glassful of water, to be used as a gargle each time.

After gargling, a teaspoonful in a tablespoonful of water, more or less, to be slowly swallowed, and repeated every three to six hours.

This simple plan of treatment may be varied by increasing or decreasing the quantity of the Perchloride of Iron. For very young children sometimes only five to ten drops, to the two ounce mixture, seldom exceeding the drachm called for in the prescription, for adults, preferring to increase or decrease the frequency of its adminisiration. The Liq. Ferri Perchlor. is not the muriated tincture of iron, though the preparations are both perchlorides. I do not get the same results from the tincture as I get from the solution, and, therefore, do not now ever prescribe the tincture, though I have thought druggists sometimes use it under the impression that they are identical. I have often to write on prescriptions, "not tincture of iron." I have no doubt there are cases occurring in this and other localities requiring other agents, as Quinia, milkpunch, etc., but within the limits of my practice I do not observe them. Diet, bread and milk, to be taken whether there is appetite or not.

I think it a little remarkable that I get very small children to use this gargle; not unfrequently they ask for it when the time draws near to take it. They say it makes their throats feel better. It saves them from having brushes or swabs put in their throats, a proceeding to which they never fail to object."

3. *Salicylic Acid as an Antipyretic.* Dr. C. A. Ewald. (*Practitioner,* March, 1876.)

Dr. Ewald (assistant physician to Prof. Frerich's Ward in the Charité Hospital, Berlin) writes on this subject, after having administered between 300 and 400 doses of salicylic acid. He says that there are eight or nine authors in Germany writing to-day on the action and therapeutical uses of this acid. However they may differ on other points, they are all united in asserting that this agent has great antipyretic power. Where reduction of temperature is sought, the administration and action of salicylic acid must be the same. The best preparation is the sodium salt (readily soluble in water), in view of the fact that this salt is doubtless produced in the blood when the pure acid is used. The dose should be large, as small or divided doses have little no no effect. Seventy-five grains should be at once administered, and if no reduction of temperature follows in four or five hours, a second, or indeed a third similar dose should be given. Two hundred and seventy-five and even three hnndred grains have been given by the doctor in the course of twenty-four hours without any ill effect. Where malaise or vomiting follows, three or four drops of chloroform internally will remove them.

After one hundred single doses of salicylate of sodium, given at midday, in cases of typhoid fever of nearly equal severity, there was almost immediately a fall of temperature, the maximum result being reached in most cases in from four to five hours, and rarely, in from eighteen to twenty-four hours. This fact was fully established by thermometric observations made every ten minutes in the axilla and rectum. This reduction of temperature was observed in eighty per cent. The results prove the superiority of salicylic acid over all other known antipyretics. "Within fifteen minutes, or even less, after the administration, a copious perspiration breaks out, first on the face, then on the thorax, abdomen and the rest of the body, accompanied by a redness of the skin, more especially that of the face, and may be so copious that the patients may lose 500 to 750 grammes of water" (a pint to a pint and a half). The defervescence begins with the perspiration, though the two do not always continue *pari passu.* Generally the pulse, respiration and intestinal tract are unaffected. Where lesions are produced on the latter, they have arisen from impurities along with the drug, as carbolic acid. The acid is eliminated from the kidneys and appears as *salicyluric* acid. Tinnitus aurium, vertigo, hallucination in one case, and, where the sweating has been excessive, partial collapse, have occasionally been observed. Where this agent has been administered to feeble patients, analeptic and stimulating remedies have been given at the same time to forestall collapse. The *modus operandi* of this drug is whollly unsettled. Its beneficial use in polyarthritica rheumatica is universally attested. "Indeed, one may say, with certainty, that in *many cases* (of rheumatic fever) after three or four doses, or even after five or ten grammes, (75 or 150 grains) not only is the fever reduced but the *articular pains* also are dispersed, so that in a FEW DAYS *acute cases may be looked upon* AS CURED. Whether, however, the tendency to relapses and inflammations of serous tissues is lessened is doubtful, and, indeed, from my own experiences must be negatived."

4. *Hypodermic Injections of Cold Water for the relief of Pains.* S. H. DESSAN, M. D. (*N. Y. Med. Jour.*, June, 1876.)

The notes of seven cases of relief from the articular pains of acute rheumatism are given somewhat in detail. Nothing but cold water was used hypodermically. The stereotyped result in every case was *immediate* relief from pain. The injection was made about the painful part. The larger the amount of water used, the longer was the relief. When only ten drops were used the relief was of very short duration. When a syringe full of water or more was used, the relief was very prolonged. The writer reports only rheumatic pains relieved thus.

5. *Some Bad Effects of Salicylic Acid in Acute Rheumatism.* JOSEPH G. RICHARDSON, M. D. (*Phil. Med. Times*, May 13, 1876.)

The reporter records four cases in which this agent, so lavishly lauded of late in rheumatism, was followed by some adverse symptoms. In the first case 140 grains of salicylic acid were administered in 120 hours, "when it produced *nausea* and was discontinued, without any apparent effect upon the rheumatic complaint having resulted." In the second case, 110 grains, given in 72 hours, produced a reduction of temperature to 96½°, F., *the pulse and heart beat becoming intermittent.* The pain and swelling were entirely abolished, and the patient received quinine, beef tea and punch freely, and was soon discharged. In the third case a hyper-defervescence resulted from taking 45 grains in 3 days. In the fourth case, 55 grains were administered in 48 hours, to a man 51 years old, an old rheumatic, on the *tenth* day of the attack. His pains were much relieved, but this "favorable result was attended with profuse perspiration, rapid reduction of temperature and marked diminution of the frequency and force of the pulse;" he also complained of great prostration, and was slightly delirious upon waking from sleep. In all these cases the tendency toward alarming prostration seems to be decided.

6. *Toothache.* W. B. HOLDERNESS, M. D. (*Practitioner*, 1876.)

A paste, made in the palm of the hand, by dropping on to a pinch of soda bicarbonate as much laudanum or wine of opium as the soda will take up, put into a cavity of the carious tooth, will often relieve the pain wholly.

7. *Poulticing in Hæmoptyses.* NOEL GUENEAU DE MUSSY. (*Union Médicale*, May 4, 1876.)

In an attack of hæmoptysis, the writer recommends poultices between the shoulders, at a temperature of about 122°, renewed every four hours.

8. *Amyl nitrite in Nervous and Mental Diseases.* PROF. PICK. (*Irrenfreund*, 2, 1876.)

The physiological effect of an inhalation of amyl nitrite is a dilatation of the arteries, especially of the head; a diminution of the arterial pressure, and an acceleration of the action of the heart. The psychological effect of the remedy is manifested by a hilarious mood of the patient. The effect lasts but a few minutes, and leaves no unpleasant sensations unless an impure preparation was used. The increased frequency of the contractions of the heart is due to the diminution or total abolition of the influence of the pneumo-gastric nerve on the functions of the heart. The dilatation of the arteries is caused by the direct influence of the amyl nitrite in the blood on the muscular tunic of the vessels. The amyl nitrite has successfully been employed in all those nervous and mental diseases which are caused by muscular contractions of the arteries or by an excessive arterial pressure.

*Hemicrania*, attended by hard pulsating temporal arteries, and by extreme paleness of the face, was quickly relieved by the inhalation of amyl nitrite.

*Epilepsy*, caused by an anæmia of the brain, as the result of arterial spasms, yielded readily to the inhalation of amyl nitrite. If there was an "aura" preceding the convulsions, the attack could almost always be prevented by an inhalation. Brunton first recommended the amyl nitrite for *angina pectoris*, and its favorable influence on this affection has since been confirmed by many writers.

As to its therapeutic value in melancholy the statements are very conflicting. Meynert claims to have obtained favorable results from its use, while others do not think their patients have been benefited by it.

9. *Croton Chloral.* EMMERT. (*Klin. Monatsbl. f. Angenheilk. Memorabilien*, xxi., 2.)

Emmert's experiments lead to results directly opposed to Liebreich's. The croton chloral he experimented with was obtained from Liebr., and employed in the doses recommended by it. "In the most cases four grammes were given. In all cases I observed a blushing of the face, and at first an acceleration, then a retardation of the respiration; the rate of the pulse varied between 60 and 90. Only in one single case there was an undoubted effect of the remedy; it was dubious in all the others. An iridectomy was performed on a very nervous patient, who, under the influence of croton chloral, grew very hilarious and laughed at the operation. A few days later the same operation was performed on the same patient's other eye; but this time no chloral was given and the patient experienced great pain." E.'s conclusions are: 1. Croton chloral may be given safely in doses of six grammes; 2. It is unreliable in its effects; 3. That it does not procure any sleep; 4. It does not cause a complete anæsthesia of the trifacial nerve; 5. It diminishes somewhat the nervous irritability.

10. *Specific against Hydrophobia.* GRZYVALA. (*British Md. Jour.*, April, 1876.)

The writer claims for XANTHIUM SPINOSUM antirabic properties. Its efficacy has been tested in one hundred victims bitten by rabid animals, of whom he lost none. Some astonishing instances of the marvelous power of this drug are given, two of which are appended. Twelve persons of one family had been bitten by a mad wolf. Six of this number were admitted into the hospital of Olschanka, Government of Podolia, district of Balta, and were treated with this drug, and all recovered. All of the others, treated with the actual cautery and the daily use of *genista tinctoria* died with hydrophobia in from twelve to sixty days. Thirty oxen had been bitten by a mad wolf; five of them died hydrophobic. The remaining twenty-five were treated with XANTHIUM SPINOSUM and recovered. Of the dried leaves, powdered, the dose for an adult is nine grains, thrice daily. For children under that age, half that dose. For the animals above alluded to, the dose was three ounces daily, given in bran. Too warm a welcome to this new aspirant for the honors of specificity against hydrophobia cannot be extended. The trustworthiness of Dr. Grzyvala is vouched for by Prof. Guber, of Paris.

(XANTHIUM SPINOSUM, or CLOT WEED, is a native of the United States as well as Europe, growing in the fields and roadsides from Massachusetts, to Pennsylvania and Georgia. It is a plant growing about one foot high very conspicuously armed with straw-colored spines, and possessed of distinctly sudorific properties.—ED. JOUR. AND EXAM.)

11. *Butyl Chloral*, (*Crotonchloral.*) O. LIEBREICH. (*Deutsche Med. W.*, Centralbl. f. Chir. 18, 1876.)

The writer experimented on rabbits to determine the physiological effect of the butyl chloral. The animals first fell into a profound sleep and exhibited a complete anæsthesia of the head, while any stimulation applied to the extremities still brought out the reflex actions. The anæsthesia then gradually extended from the head over the whole body, when the reflex movements could be elicited no longer. When death occurred the respiration always ceased prior to the action of the heart, and the autopsy conclusively proved the animals had died of suffocation. This shows a remarkable difference in the action of the croton chloral and hydrate of chloral; for the latter remedy kills the animals by paralysis of the heart, and the artificial respiration fails to restore the life, while this can very successfully be employed in the case of death from croton chloral. L. has also studied the effect of the croton chloral on men; and here, too, the anæsthesia of the head was complete, while the reflex action of the limbs was not abolished. For this reason L. thinks that the croton chloral might be used with advantage for operations about the head, especially in such cases where the inhalation of an anæsthetic is not well tolerated. As to the administration, L. prescribes the following formula:

℞ Butyl Chloral 5 to 10, ʒ one to two; Glycerine 20, ʒ v; Water 130, ℥ vi. Shake the bottle before use. The patient takes one tablespoonful; after five minutes another, and ten minutes later a third one.

Butyl chloral, in doses of one to three grammes, may be given also for toothache.

12. *The Wet Bandage* vs. *Tartar Emetic.* B. H. WASHINGTON, M. D., of Augusta, Georgia. (*Nashville Journal of Med. and Surgery*, May, 1876.)

Heat kills more pneumonia patients than degenerative changes of lung structure. The chief heat abrogators of to-day are tartar emetic and quinine. The writer says: "If any one will use the wet bandage in the treatment of pneumonia, to reduce the superabundant heat, he will never feel any inclination to try tartar emetic again." To use the bandage, "the patient's clothes should be slit down so that the attendant can change the bandage every four or five minutes without disturbing him; after the intense heat is well reduced it will not be necessary to change it so often." The temperature of the water at first should not be low enough to shock the patient. With robust patients colder water can generally be used; with delicate patients and children it is best to use tepid water throughout. The bandage should be covered with a dry one, and then no fears need be entertained about damaging the invalid. The urgency of the cough disappears greatly under the use of the bandage. Throughout the treatment the terrible heat of this disease can be kept subdued all the time by the wet bandage. Other measures recommended in conjunction with the water application are quinine and an opiate cough mixture at night. With this therapeutic combination tartar emetic is wholly unnecessary.

13. *Topical Treatment of Chronic Dysentery.* M. DILLS, M. D., of Carlysle, Ky. (*N. Y. Med. Jour,* April, 1876.)

The reporter narrates three cases of topical treatment of dysentery, followed by cures. They were of several months standing each. The first case, that of a girl fourteen years old, was of six months duration. She was in a very low condition, with a pulse 130 and scarcely perceptible, skin covered with a clammy sweat. Her body had emitted a cadaveric odor for several days; death seemed inevitable. After etherization, a bivalve speculum was introduced into the rectum and the "mucous membrane was found highly inflamed and studded over with small yellowish ulcers, which, on slight pressure, emitted a colored fluid." Silver nitrate was freely applied to every part of the bowel, as high up as could be reached with the aid of a retractor. This application was followed by an ability to control the bowel. The appetite was improved, strength increased, and recovery of the vital powers was very speedy. Daily injections of carbolic acid solution (one part to eight of water,) were used. In two weeks the patient made a complete recovery. The other two cases were similarly treated and recovery followed.

14. *Sulphurous Acid in Enteric Fever.* J. WESLEY BOTKIN, M. D., Morrisonville, Ill. (*Phila. Med. and Surg. Reporter,* May, 1876.)

Thirty cases were treated with sulphurous acid, in doses ranging from three to fifteen drops, in lemonade, every four hours. Only one patient of this number died; this one patient "was a fragile girl, whose life was gradually wasting away with consumption, but she recovered, then relapsed and died." The writer thinks the acid acts as a specific upon the fever poison, arresting at once its further development and thus exterminates the fever. Amelioration ensues at once, and in a very few days the patients, under the influence of this agent, are convalescent. Within twenty-four hours the tongue becomes moist and commences to clean; the diarrhœa is speedily arrested, the tympanites subsides, the pulse slows and grows stronger, the digestive faculty speedily asserts itself and the patient is soon out of danger.

15. *Treatment of Diphtheria by Clysters.* W. H. VAIL, M. D. (*N. Y. Med. Record*, May 13, 1876.)

One case is reported — "a true malignant case." Patient was twenty-two months old. Refusing to swallow, the rectum was used. Every four hours during the first week the remedies and food, with brandy, were injected together. When they were dejected, the clyster was immediately repeated, with ten drops of laudanum added, and in every instance the second injection was retained. Alvine dejections were normal and well formed. As the severe symptoms abated the lavements were used every six hours. The rectum was used thus for three weeks. Recovery took place.

16. *Anaphrodisiac Properties of Tobacco.* MARTIN-DAMOURETTE. (*Jour. de Méd. et de Chir.*, May, 1876.)

The anaphrodisiac properties of tobacco have long been known, as Foussard has well shown in his work, and have induced its use in the numerous convents of Italy. When the cause of impotencc is obscure, it is well for physicians to remember this noxious property of tobacco, as the following cases observed by the author show:

A young man who smoked more than twenty cigars per day, complained of loss of digestive power, weakness, feeble memory and impotence. As he was about to marry, he consulted a physician. The latter, aware of the habits of his patient, ordered him to discontinue the use of tobacco, which was followed by restoration of the genital function.

A young physician had complete genital frigidity, for which he had taken strychnia till he consumed thirty-six centigrammes daily, without either injurious or remedial results. The author found upon investigation that he smoked cigarettes only, but used them constantly throughout the day. His muscular vigor and power of resisting fatigue were thus sensibly diminished; and the author concluded that the incredible tolerance of strychnia was due to profound paresis of the motor nerves, occasioned by the excessive and gradually increasing use of tobacco. By abandoning its use, this patient was perfectly relieved, without resorting to medicine or hygiene.

A young and robust student of the polytechnic school became an inspector at a tobacco manufactory. He soon experienced considerable loss of genesic power and finally became impotent. Both the patient and physician agreed as to the probable cause of the disease, and after vainly making trial of other remedies, the inspector of tobacco engaged in another business, when he speedily recovered his generative power.

17. *Treatment of Diabetes Mellitus by Phenic Acid.* (*Bordeaux Médical*, April, 1876.)

In accordance with the latest theories respecting organic ferments, phenic acid is supposed to influence diabetes mellitus by reason of its well known property of antagonizing the action of ferments. Whatever may be the mode of its operation, its administration is readily effected, is unaccompanied by danger, and is serviceable when ordered as follows: Equal parts of the alcoholate of mint and phenic acid are mixed, and two or three drops of the solution are taken morning and evening in a cup of any agreeable infusion.

18. *To make Castor Oil Tasteless.* (*Amer. Practitioner.*)

Squeeze half an orange into a glass, and pour the oil upon it; then, avoiding disturbing the liquids, squeeze the juice from the other half of the orange over the oil. The contents of the glass can then be "tossed off" without the least perception of the oil flavor.

19. *Nitro Glycerine.* A. J. MINOR, M. D. (*Amer. Psycholog. Jour.*, 1876.)

Therapeutists have long ago determined the healing properties of hydrocyanic acid, woorari, veratria, strychnia, aconitia, and very many other terribly destructive agents; but it is only recently that the therapeutic effect of a diminutive *earthquake*, in the shape of nitro-glycerine, has been recorded. Dr. Minor declares that this agent, in astonishingly minute quantities, will quickly develop a violent headache in a person of sanguine temperament; only as much as can be collected on a "pin's point dipped in the liquid, and the pin shaken to remove any adherent particle in the shape of a drop," being required to produce the above named effect within five minutes. Its supposed action is paresis of the cerebral vaso-motors, and its consequent congestion. One writer regards it as being "highly indicated as a prophylactic in epileptic seizures." It differs from amyl nitrate in being more continuous in its action. It seems to be indicated in all spasms of the cerebral vessels. It *has* cured cases of neuralgia, but under what conditions cannot be clearly defined.

20. *Intestinal Atony.* MCSHERRY. (*Canada Med. Record.*)

For asthenic constipation, the writer recommends ergot and phosphorus; the latter apparently enhances the action of the former. Ergot, it is well known, is a vaso-motor stimulant; vascular contraction follows its ingestion, and this partial anæmiation produces muscular contractions; hence the increased peristalsis. This formula is recommended:

℞ Fl. Ext. Secal. Cornut., ............................... ℥ vij.

Acid Phosphorici Diluti,............................... ℥ j.

M. S. Teaspoonful three times a day.

21. *Odorless Iodoform.*

Ether dissolves iodoform and robs it of its unpleasant odor. Applying this solution of it, leaves it in a uniform SMELLLESS coating on the surface receiving it after the evaporation of the ether. *N. Y. Med. Record* and *Cincin. Clinic* please take notice.

22. *Cold in the Head.* FERIER. (*Doctor*, May, 1876.)

Following prescription recommended to be taken as a snuff. One quarter to one-half may be taken in 24 hours, with assurance of certain relief from coryza:

℞ Hydrochlorate of Morphia,............................gr. ij.
Acacia Powder ...........................................dr. ij.
Trisnitrate of Bismuth ....................................dr. vj.
Mix well.

23. *Danger of Chloroform in Anal Fissure.* NICAISE. (*Gazette Médicale.*)

Patients with the "intolerable" variety of anal fissure seem to possess a nervous system unusually sensitive to the dangerous effects of chloroform. Forced dilation of the sphincter being the almost exclusive means of treatment of to-day, anæsthetization is necessary. The writer mentions three patients afflicted with fissure, who were chloroformized and very speedily passed into an alarming state of resolution, with thorax immovable and pulse nearly extinct. Dilatation being immediately performed, the patient, in one instance, came too almost at once. In the other two cases, means of restoring had to be continued for a long time. But small amounts of chloroform were used in each instance. The exalted sensitiveness of the nervous system induced by the terrible suffering incident to anal fissure, is the probable explanation of the remarkable cases here alluded to.

24. *Gelseminum.* CARL HERTZKA ET A. JAURASZ. (*Centralbl. f. d. Med. Wessensch.*, 1875, Nrs. 47 et. 31.

Pain and weakness in both arms, of two years standing, arising from piano playing, were cured by taking 8 drops of tincture of gelseminum thrice daily for three weeks. In cases of neuralgia of the tregeminal, supraorbital, brachial and sciatic nerves, this drug, in 5-20 drops of the tincture, thrice daily, has been successfully used. In some cases it fails utterly, but in a larger proportion of cases of the neuralgias it can be reliably used.

25. *Treatment of Sunstroke with Hypodermic Injections of Quinine.* A. R. HALL, Surg. British Army. (*Practitioner*, 1876.)

All cases of insolation treated with subcutaneous administration will doubtless receive the *best* chances of recovery. Old army surgeons in British India, where the best opportunity for observing this malady is offered, say the effect of quinine thus applied "may be described as *magical.*" Heat is, at first, a vaso-motor stimulant—too long continued in, this stimulation, becomes exhaustion, and then the peculiar condition of the system following is denominated *sunstroke.* The vaso-motor control over the vessels is lost, the cutaneous vessels are turgid with blood, and the sweat glands have apparently lost their power. Theoretically, quinine stimulates the vaso-motors and thus produces capillary contraction; and the peculiar train of morbid circulatory symptoms is broken up, and convalescence sets in. However, whatever the *theory* may be, the practice of putting three to five grains of quinine under the skin is productive of speedy recovery in nearly all cases.

26. *Hyposulphite of Soda in Diphtheria.* Tamborlini. (*Raccoglitore Medico.; Jour. de Med. et de Chir. Prat.*, April, 1876.)

The remedy not only tends to diminish the temperature but to destroy the cryptogam of the false membrane. The hyposulphite of soda is given in doses of from 6 to 20 grammes in from 100 to 300 grammes of distilled water, to which 30 grammes of syrup of orange peel are added. At the same time a gargle is administered, containing 40 grammes of the hyposulphite of soda in 400 of distilled water. The diet should consist of eggs, soup and wine. During convalescence the prolonged use of lactate of iron is recommended. Dr. Tamborlini reports numerous successful cases thus treated.

27. *Convulsions arrested by the Sinistro-Lateral Posture.* F. J. Brown, M. D. (*Practitioner*, 1876.)

Mr. Bader first pointed out to the profession the advantages of putting the patient on the left side when threatened with danger under chloroform inhalations. Knowing the good effects of this procedure, Dr. Brown applied it in case of a man with Bright's disease, in a urecmic convulsions, and the latter ceased instantly. Again he resorted to the same expedient in a man sieized with unilateral (right) convulsions, whose consciousness and speech were intact: he had been convulsed ten minutes, when the doctor arrived and immediately turned him upon his right side, and the convulsive action ceased in ten or fifteen seconds. What does this mean? Similar successes are greatly desired to confirm a startling fact—if this postural treatment resulting curatively *be* a fact.

